# Oxidative DNA damage in diabetic and mild gestational hyperglycemic pregnant women

**DOI:** 10.1186/1758-5996-7-1

**Published:** 2015-01-15

**Authors:** Rafael Bottaro Gelaleti, Débora Cristina Damasceno, Paula Helena Ortiz Lima, Daisy Maria Favero Salvadori, Iracema de Mattos Paranhos Calderon, José Carlos Peraçoli, Marilza Vieira Cunha Rudge

**Affiliations:** Botucatu Medical School, UNESP - Univ Estadual Paulista, São Paulo, SP Brazil; Instituto Dante Pazzanese de Cardiologia, São Paulo, SP Brazil; Departamento de Ginecologia e Obstetrícia, Faculdade de Medicina de Botucatu, UNESP, Distrito de Rubião Júnior s/n, CEP. 18618.000 Botucatu, São Paulo Brazil

**Keywords:** Diabetes, Pregnancy, Mild gestational hyperglycemia, Genotoxicity, Oxidative DNA damage

## Abstract

**Background:**

Pregnant women with mild gestational hyperglycemia present high risk for hypertension, obesity and hyperglycemia, and appeared to reproduce the model of metabolic syndrome in pregnancy, with hyperinsulinemia and insulin resistance. Our clinical studies showed that mild gestational hyperglycemia or gestational diabetes are related to similar adverse maternal and perinatal outcomes. Hyperglycemia and other factors associated with diabetes generate reactive oxygen species that increase DNA damage levels. The aim of this study was to evaluate oxidative DNA damage in lymphocytes of pregnant women with diabetes or mild gestational hyperglycemia.

**Methods:**

The study included 111 pregnant women distributed into three groups based on oral glucose tolerance test (OGTT) and glycemic profiles (GP), as follows: Normal OGTT and GP (control group); Normal OGTT and abnormal GP (mild gestational hyperglycemia group); Abnormal OGTT and GP (diabetic group). Maternal blood samples (5–10 mL) were collected and processed for determination of oxidative DNA damage by the comet assay, using Fpg and Endo III enzymes. Urine samples were also collected for determination of 8-OHdG concentrations by ELISA.

**Results:**

Subjects in the diabetes group presented increased amount of oxidized purines, while mild gestational hyperglycemia women presented with increased oxidized pyrimidines, compared to the control group.

**Conclusion:**

Gestational, overt diabetes and mild gestational hyperglycemia, were all related to increased oxidative DNA damage. Diabetic pregnant women showed increased level of oxidative DNA damage, perhaps mainly due to hyperglycemia. On the other hand, oxidative DNA damage detected in women with mild gestational hyperglycemia might be associated with repercussions from obesity, hypertension and/or insulin resistance. Interestingly, the type of DNA base affected seemed to be dependent on the glycemic profile or oxidative stress.

**Electronic supplementary material:**

The online version of this article (doi:10.1186/1758-5996-7-1) contains supplementary material, which is available to authorized users.

## Introduction

The Hyperglycemia and Adverse Pregnancy Outcome (HAPO) Study has demonstrated significant perinatal risks at levels of maternal hyperglycemia below values that are diagnostic for diabetes [1]. Furthermore, this study has confirmed our previous findings showing that glycemia lower than GDM (Gestational *Diabetes mellitus*) is also related to adverse perinatal outcome [2]. The new criteria for diagnosis of GDM identified a group of women that were previously classified as normal according to the 4th International Workshop Conference criteria [3]. This group has metabolic characteristics and pregnancy outcomes resembling women who would have been considered to have GDM by previous criteria.

Since 1990, Rudge *et al.*[4] have combined two parallel diagnostic tests, 100 g Oral Glucose Tolerance Test (OGTT) and Glycemic Profile (GP), to characterize the mild gestational hyperglycemia (MGH) pregnant group, women with positive screening for GDM, negative diagnosis for GDM but with hyperglycemia detected in the GP. In diurnal GP the fasting plasma glucose, 1–h over a 2-h period post-ingestion of general diet are collected. The MGH group represented 13.8% of positive screened pregnant women for GDM and, added to 7.0% of pregnancies complicated by diabetes, increase the occurrence of hyperglycemic disorders in pregnancy to about 20% [5]. These patients were at high risk for hypertension, obesity and hyperglycemia, and appeared to reproduce the model of metabolic syndrome (MS) in pregnancy, with hyperinsulinemia and insulin resistance, which continued six weeks postpartum [6]. After 10 to 12 years of the index-pregnancy, type 2 diabetes was confirmed in 16.7% of women with MGH during pregnancy [7]. Exploratory analyses with newborn outcomes indicated 53.8% with macrosomia, a similar proportion (51.9%) to that described for overt diabetes and GDM [8]; a high perinatal mortality rate (41%) was also similar to that observed in diabetic women and was10 times greater than that of normal pregnant women [9]. These newborns may also exhibit hypoglycemic crises, hyperbilirubinemia and a high incidence of prematurity and congenital anomalies [2]. Therefore, despite being identified in the literature as low-risk, MGH in pregnancy is associated with adverse maternal and perinatal outcomes, previously classifiable as normal according to the 4th International Workshop Conference criteria. Furthermore, MGH patients represent a significant public health problem and must be reclassified as abnormal according to maternal and perinatal findings [9]. Therefore, it is crucial to understand the underlying mechanisms involved in MGH in order to detect If MGH and GDM are the same disease in different stages or are different pathologies with similar maternal and perinatal results. This is important since the stimuli to detect hyperglycemia and MGH are different. In GDM hyperglycemic levels are detected after ingestion of 75 g of glucose and in MGH hyperglycemia is detected after ingestion of a general diet.

Type 2 diabetes is the most common endocrine disorder affecting more than 5% of the population in western countries [10]. The pathogenesis of type 2 diabetes is characterized by both decreased peripheral insulin action and impaired pancreatic β-cell function [11, 12]. In the majority of the prediabetic population, the earliest abnormality is insulin resistance that precedes the development of glucose intolerance [13]. Initially, the pancreas attempts to compensate for insulin resistance through increased insulin secretion. When the pancreas is unable to maintain a sufficient hyperinsulinemic response, frank diabetes ensues as a consequence of progressive loss of β-cell function. Patients with type 2 diabetes are also characterized by reduced β-cell mass as compared with weight-matched non-diabetic subjects [14].

Animal models have confirmed the human epidemiologic findings and elucidated potential programming mechanisms of MGH, that include altered organ development, cellular signaling responses, and epigenetic modifications. Using an animal model, we have shown that rats with mild (glycemia from 120 to 300 mg/dL) or severe (glycemia > 300 mg/dL) diabetes and their newborns exhibited increased oxidative DNA damage detected by the comet assay [15]. Increased levels of DNA damage have been previously observed in leukocytes of type1 diabetes and type 2 diabetes patients [16–18]. Dinçer *et al.*[17] have confirmed increased DNA damage and sensitive FAP glycosylase restriction sites in type 1 diabetes*,* and Pitozzi *et al.*[18] have detected increased oxidative DNA damage in polymorphonuclear leukocytes from type 2 diabetes. Other clinical and experimental investigations have also shown the presence of DNA oxidative damage in diabetic patients [19–21]. Diabetes and hyperglycemia can be sources of DNA damage via the oxidation of DNA bases and sugar-phosphate binding sites [22].

Therefore, since our clinical studies showed that GDM or MGH are related to similar adverse maternal and perinatal outcomes, and the animal models demonstrated increase oxidative DNA damage in moderate and severe diabetic rats, we hypothesized that even though not diagnosed with diabetes, MGH pregnant women might have increased levels of this genotoxic event. If this hypothesis is correct, overt diabetes, GDM or MGH can each contribute to DNA damage and represent different stages of the same pathology. The aim of the present study was to evaluate oxidative DNA damage in lymphocytes from pregnant women with a wide range of glucose tolerance. Using different restriction enzymes, this manuscript demonstrated different answer according to glycemic variation and others factors associated to diabetes in pregnant women with diabetes or mild gestational hyperglycemia.

## Research design and methods

After approval by the Research Ethics Committee of Botucatu Medical School number (OF 545/2004) the present investigation was conducted at Perinatal Diabetic Research Center - Botucatu Medical School, UNESP, Brazil. This study included 111 pregnant women. The pregnant women were assigned to participate if they presented with a fasting glycemia level ≥90 mg/dL and/or risk factors for developing gestational *Diabetes mellitus* between the 24th and 28th week of gestation. A 75 g oral glucose tolerance test (OGTT) and a glycemic profile were performed; the cutoff values for the OGTT were those proposed by Carpenter & Coustan (fasting ≥95 mg/dL; 1 h ≥ 180 mg/dL; 2 h ≥ 155 mg/dL;) and for the glycemic profile, those proposed by Gillmer *et al.* (fasting ≥ 90 mg/dL and/or postprandial ≥ 130 mg/dL) (Additional file 1 (flowchart)). After these procedures the patients were classified into three groups:

Normal OGTT and glycemic profile (normoglycemic or control group) n = 41

Normal OGTT and abnormal glycemic profile (mild hyperglycemic group-MGH) n = 24

Abnormal OGTT and glycemic profile (diabetic group) n = 46. (Rudge *et al.*[4]).

At screening, maternal characteristics such as age, body mass index (BMI), diabetes and hypertension were recorded. Hypertension was considered when a systolic blood pressure >140 mmHg and/or a diastolic blood pressure > 90 mmHg, on at least two occasions at least six hours apart, was detected [13]. For maternal hyperglycemia regulation, pregnant MGH and GDM women were treated with diet, physical exercise, and insulin therapy (if necessary) after the diagnosis, and women with type 2 diabetes were treated since the beginning of pregnancy [23, 24].

Inclusion criteria were defined: (a) classified in the study groups, (b) gestational age of entry into treatment’s protocol of 30 weeks for MGH and 20 weeks pregnant for type 2 DM (c) prenatal care and birth on the service, (d) consent form signature. Exclusion criteria were multiple pregnancies, fetal malformations, birth before 34 weeks and type 1 diabetes.

The glycemic mean was calculated by the arithmetic mean of plasma glucose measured in all GP performed at diagnosis (ND group) and the control of treatment (MGH and diabetic groups). Plasma glucose was measured by the glucose oxidase method (Glucose - Analyzer II Beckman®, Fullerton, California, USA) and the body mass index (BMI) was calculated by body weight divided by the square of height.

From 34 weeks of gestation and before the onset of labor, maternal blood samples (5 to 10 mL) and urine samples were collected. The blood samples were collected in Vacutainer tubes with EDTA and the urine samples in collector tubes. The collected blood samples were immediately processed for determination of oxidative DNA damage by the comet assay and the urine samples were stored in a freezer at -80°C for the measurement of 8-OHdG and creatinine levels.

To evaluate oxidative DNA damage, the comet assay was performed acoording to the protocol described by Collins *et al.*[19]. Briefly, maternal lymphocytes were isolated using a Ficoll® gradient, and 20 μl were mixed with low melting point (LMP) agarose (120 μl), placed on a precoated slide with normal melting point (NMP) agarose, and immediately covered with a coverslip. The slides were left at 4°C for 10 min to solidify the agarose. The coverslip was gently removed and the slides were immersed in an ice-cold freshly prepared lysis solution (2.5 M NaCl, 100 mM EDTA, 10 mM Tris, with 1% Triton 100-X, and 10% dimethylsulphoxide added just before use). Afterwards, the slides were washed with cold PBS (phosphate buffered saline) buffer and 1X FLARE (Fragment Length Analysis using Repair Enzymes) buffer and placed in a lined container. The enzymatic treatment was performed following the methodology described by Collins *et al*. [25]. The formamidopyrimidine DNA glycosylase (Fpg) and endonuclease III (Endo III) enzymes were used to detect oxidative damage in purine and pyrimidine bases, respectively. After enzymatic treatment, the slides were covered with coverslips and kept at 37°C for 45 minutes. The coverslips were removed and slides placed on a horizontal electrophoresis unit filled with fresh electrophoresis alkaline buffer (300 mM NaOH and 1 mM EDTA, pH > 13). The alkali unwinding duration was 40 min. Electrophoresis was conducted at 4°C for 30 min at 25 V/cm and 300 mA. All steps were carried out under minimal illumination. The slides were neutralized in a buffer (0.4 M Tris at pH 7.5) and dipped in absolute alcohol for fixation. The dried slides were stained with ethidium bromide (20 μg/ml in distilled H_2_O; 50 μl/slide), covered with a coverslip and analysed in a fluorescence microscope connected to a computer-based analysis system (Comet Assay IV, Perceptive Instruments, UK) to determine the extent of DNA damage. Results were expressed as tail intensity (% DNA in the comet tail). One hundred randomly selected nucleoids (50 from each of two replicate slides) were scored per blood sample. Positive controls consisted of lymphocytes treated with H_2_O_2_ (200 μM, 30 minutes) [26].

The 8-hidroxy-2-deoxi guanosina (8-OHdG) dosage was determined by ELISA according to the manufacturer’s protocol (8-OHdG Kit _EIA, Cayman Chemical Company). The creatinine measurement was performed by spectrophotometry (Creatinine assay Kit, Cayman Chemical Company). After 8-OHdG and creatinine determination, the mass ratio was performed between the 8-OHdG concentration and creatinine levels.

Analysis of variance (ANOVA), followed by the Tukey’s multiple comparisons test, was used for the characteristic endpoints of the study population, and the ANOVA followed by Student Newman-Keuls test for 8-OHdG dosages. To analyze the significance (p < 0.05) of oxidative DNA damage, Gamma distribution was applied.

## Results

Table 1 shows the characteristics (age, body mass index, glycemic mean, glycated hemoglobin and blood pressure) of the study population. Age and body mass index (BMI) did not differ among the groups. Higher glycemic means were observed in both the MGH and diabetic groups compared to the control group. The glycemic mean in women of diabetic group was higher than in subjects in the MGH group (p < 0.05). Glycated hemoglobin was higher in diabetic group compared to the control group (p < 0.05). The MGH group had a greater number of individuals with hypertension in pregnancy compared to the other study groups (p < 0.05).

**Figure 1:**
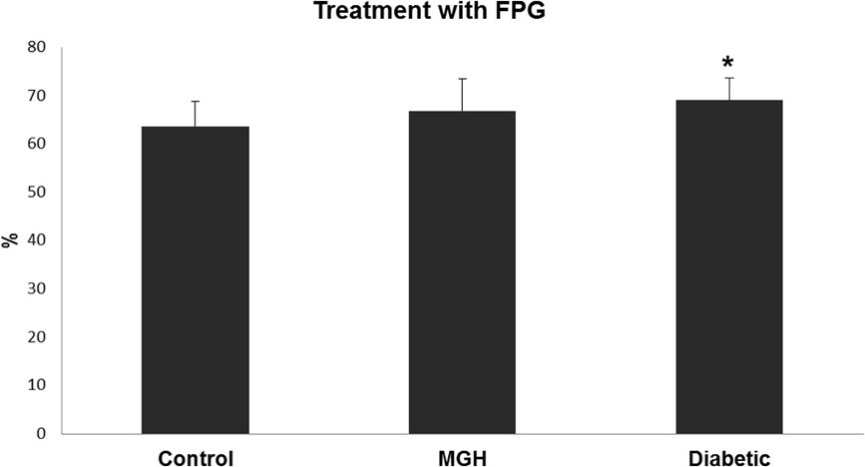
**Oxidative DNA damage levels after treatment using enzyme formamidopirimidine glycosylase (Fpg) in the study groups.** Data presented as mean ± standard error of mean. *p < 0.05 – significant difference compared to the control group (Gamma distribution).

**Figure 2:**
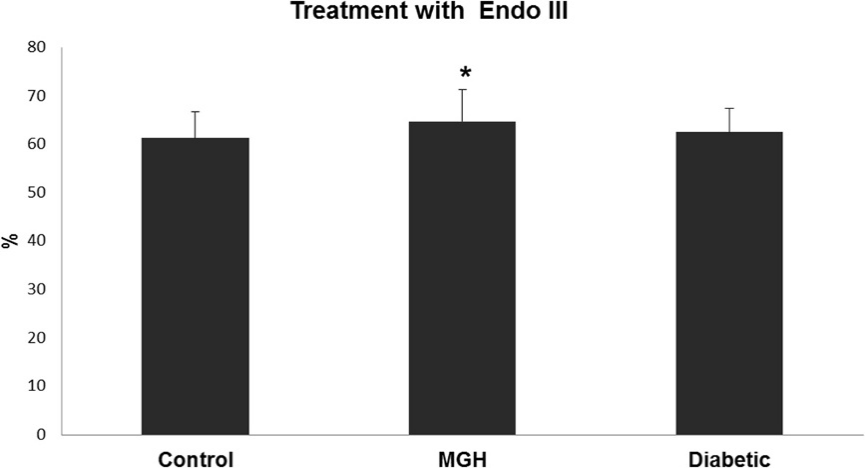
**Oxidative DNA damage levels after treatment using enzyme endonuclease III in the study groups.** Data presented as mean ± standard error of mean. *p < 0.05 – significant difference compared to control group (Gamma distribution).

**Figure 3:**
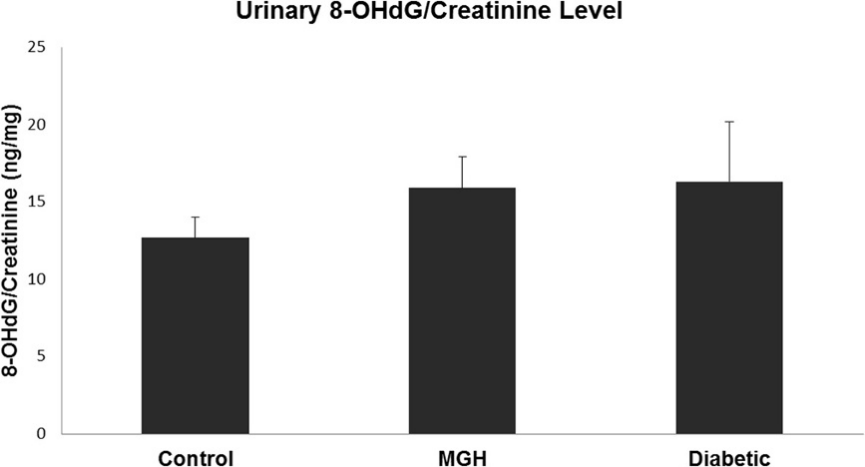
**8-OHdG concentrations (ng/mg creatinine) in the study groups.** Data presented as mean ± standard deviation of mean (Tukey’s multiple comparison test). p > 0.05 - No significant difference.

Our results showed an increased level of oxidized purines (FPG) (tail intensity) in lymphocytes from women with DG, compared to women in the control group (Figure 1) and an increased level of oxidized pyrimidines (EndoIII) (tail intensity) in lymphocytes from women with MGH compared to the control group (Figure 2). Regarding the concentration of 8-OHdG in urine, it was slightly increased in pregnant women with diabetes and MGH, although no statistically significant difference was detected (p > 0.05) (Figure 3).

**Table 1:** **Characteristics of the study population**

	Groups		
	Control	MGH	Diabetic
**N**	41	24	46
**Age** (years) ^§^	29.7 ± 5.5	31.5 ± 4.2	31.3 ± 5.4
**BMI** (Kg/m^2^)^§^	32.7 ± 6.9	36.0 ± 7.4	35.2 ± 6.3
**Glicemic Mean** (mg/dL)^§^	81.5 ± 9.5	97.9 ± 7.5*	111.5 ± 17.6*^#^
**HbA1C** ^§^	5.45 ± 0.53	5.74 ± 0.67	6.33 ± 0.90* ^#^
**Rate of Hypertension** (N)%))^||^	14 (34.1)	16 (66.6.)*^&^	17 (35.4)

HbA1C – Glycated Hemoglobin.

Data presented as mean ± standard deviation and number of subjects (percentage).

^*^p < 0.05 – significant difference compared to the control group (^§^Tukey’s multiple comparison test and ^||^chi-square test).

^#^p < 0.05 – significant difference compared to the MGH group (Tukey’s multiple comparison test).

^&^p < 0.05 – significant difference compared to the diabetic group (chi-square test).

When using the restriction enzyme Endo III, women in the MGH group with hypertension had higher values of oxidative DNA damage compared to those in the MGH group without hypertension (70.15 ± 8.7 vs. 53.74 ± 17.4) (p = 0.05).

Positive controls treated with H_2_O_2_ presented levels of DNA damage (tail intensity) of 77.82 ± 5.3 (mean ± standart error of the mean).

There are no reports about the ethnic differences in the comet assay and DNA damage.

## Discussion

The available literature provides insufficient information on the involvement of DNA damage in pregnant women with a wide range of glucose tolerance, diabetes and MGH. The hyperglycemia seen in type 2 diabetes is associated with increased oxidative stress and production of reactive oxygen species, both of which are factors that can induce DNA damage. Therefore, the aim of this study was to evaluate oxidative DNA damage in lymphocytes from pregnant women with a wide range of glucose tolerance. In this report, we demonstrate increased levels of oxidized purines and pyrimidines in diabetic and MGH subjects, respectively, compared to control mothers.

Our findings in the diabetic pregnant group are consistent with those reported by Collins *et al.*[19], in their classical study, showing that oxidized purines specifically reflect DNA damage caused by hyperglycemia. Therefore, Fpg-sensitive sites were more closely associated with increased glycemic levels. In our translational study, similar results were found in diabetic pregnant rats [27]. To the best of our knowledge, the current data showing high level of oxidized pyrimidines in the MGH group is described for the first time. FPG sensitive-sites were not increased in these women. This MGH group presented not only with hyperglycemia but also with insulin resistance, obesity and hypertension [28], which have already been associated with increased DNA damage [29–31]. High insulin resistance indices (HOMA-IR) can infer oxidative stress and DNA damage [32]. Yildiz *et al.*[29] and Gür *et al.*[31] showed that DNA damage caused by ROS occurs more commonly in hypertensive patients. The association among mild hyperglycemia, insulin resistance and hypertension may be related to the high level of oxidized pyrimidines presented in lymphocytes from women in the MGH group. Collins *et al.*[19] shows that Endonuclease III-sensitive sites are not correlated with glycemia; it is possible that reactive oxygen species resulting from glucose oxidation are more likely to cause guanine than pyrimidine oxidation. It appears that endonuclease III can give an indication of overall oxidative damage to DNA, resulting from a variety of diabetes-related causes, while FPG reflects specifically the damage resulting from hyperglycemia, namely 8-oxo-guanine [19].

In the present study we did not detect a significant difference in the urinary 8-OHdG concentration among the three groups. However, the values increased according to the concentration of glucose, suggesting a relationship between blood glucose and oxidative stress. Recently, Qiu *et al.*[33] showed that a single measurement of urinary 8-OHdG concentrations is not likely to provide a time-integrated measure of maternal cellular oxidative stress during the entire pregnancy. Therefore, longitudinal studies, with serial measurements of urinary 8-OHdG concentrations along with indices of insulin sensitivity and secretion across gestation, are needed to elucidate the mechanisms and pathophysiological consequences of maternal oxidative stress during pregnancy.

In conclusion, both diabetic and mild gestational hyperglycemia (MGH) pregnant women presented evidence of increased oxidative DNA damage. However, the specific DNA base that was mostly affected in the two conditions were different, purines in diabetic pregnancy and pyrimidines in MGH. Taken together, the present data underscore that hyperglycemia is an important stimulus to maternal DNA damage and even mild forms of hyperglycemia can induce measurable DNA damage and adverse consequences for pregnant women and their offspring [4, 2, 1]. Gestational dysglycemia (diabetes and MGH) identifies a group of women with higher oxidative DNA damage.

The utility of identifying the unique DNA bases mostly affected in MGH suggests that this condition is characterized by a group of patients not only with hyperglycemia but also with other clinical parameters responsible for oxidative DNA damage, indicating that regardless of glycemic intensity, affected bases and forms of treatment, hyperglycemic and diabetic groups require strict medical control in order to control hyperglycemia and other risk factors such as obesity, insulin resistance and hypertension by targeted intervention. One goal of future investigation is to identify screening tools and biomarkers that may be evident in women with MGH before the emergence of diabetes.

## Electronic supplementary material

Additional file 1: **Flowchart 1.** Subject Follow up. (TIFF 54 KB)
