# Supplementary figures and images for: Oxidative DNA damage in diabetic and mild gestational hyperglycemic pregnant women
Source: Diabetol Metab Syndr. 2015 Jan 15;7:1. doi: 10.1186/1758-5996-7-1 (PMC4373109; doi:10.1186/1758-5996-7-1)

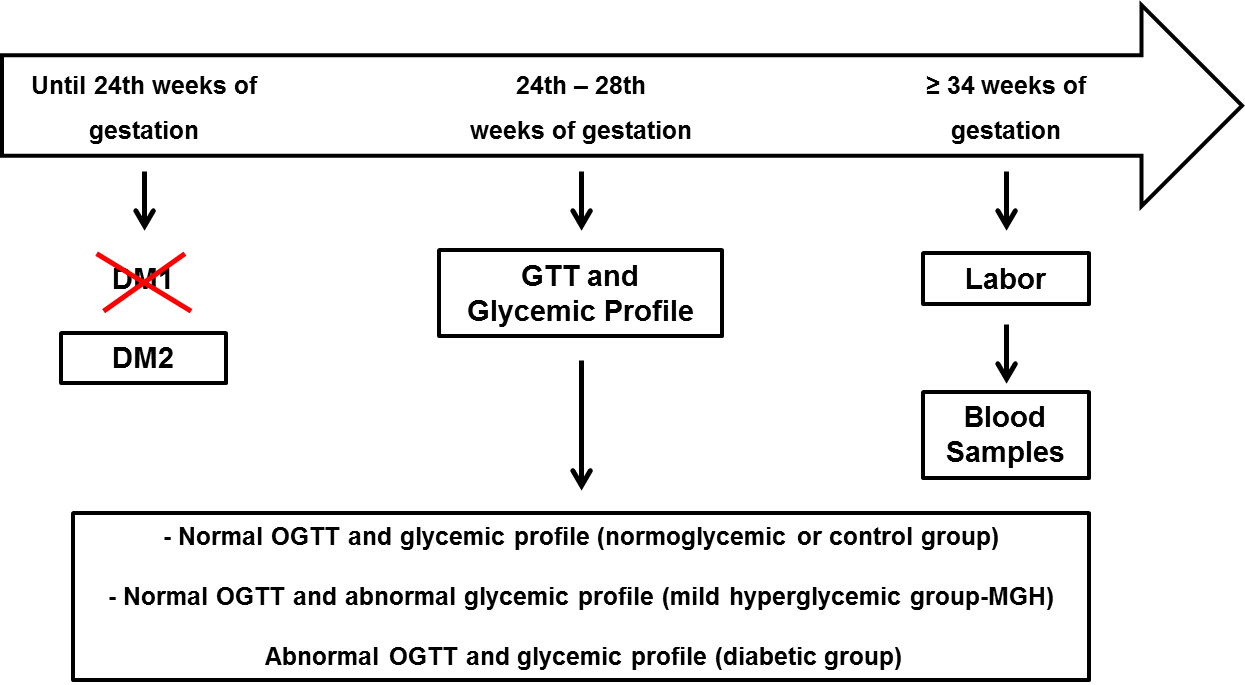

Supplement: Supplementary file 1 — Additional file 1: Flowchart 1. Subject Follow up. (TIFF 54 KB) [file 13098_2014_406_MOESM1_ESM.tiff]
